# Establishment and verification of reference intervals for blood cell analysis in extremely high altitude

**DOI:** 10.3389/fphys.2024.1383390

**Published:** 2024-10-14

**Authors:** Zhimin Yuan, Jian Zhuang

**Affiliations:** ^1^ Department of Clinical Laboratory, Shaanxi Provincial Cancer Hospital, Xi’an Jiaotong University, Xi’an, China; ^2^ Department of Clinical Laboratory, The People’s Hospital of Ali District, Ali, China; ^3^ Extracorporeal Circulation Laboratory, The First Affiliated Hospital of Xi’an Jiaotong University, Xi’an, China; ^4^ School of Mechanical Engineering, Xi’an Jiaotong University, Xi’an, China

**Keywords:** complete blood count, Tibetan, extremely high altitude, reference range, Ali district

## Abstract

**Objective:**

This study aimed to establish the reference intervals for complete blood count (CBC) in healthy adults at very high altitudes.

**Methods:**

This study recruited 4,863 healthy adults (3,598 males and 1,265 females) who received routine health check-ups at Ali District People’s Hospital from January 2019 to December 2021 through the LIS system. CBC was detected by the XT-4000i automatic cell analyzer and statistically analyzed by SPSS 23.0 software (between-groups comparison, diagnostic concordance comparison). In addition, 20 health check samples were collected in 2022 to validate the established reference intervals.

**Results:**

The CBC count showed a non-normal distribution in each item separately. The white blood cell (WBC), neutrophil (NEUT), lymphocyte (LYMPH), monocyte (MONO), eosinophil (EO), basophil (BASO), red blood cell (RBC), hemoglobin (HGB), hematocrit (HCT), mean corpuscular volume (MCV), mean hemoglobin content (MCH), and mean erythrocyte hemoglobin concentration (MCHC) levels of healthy adults were significantly different from the national clinical reference range (*p* < 0.05). There were significant differences between males and females in RBC, HGB, and HCT levels (*p* < 0.05). The new reference intervals were less consistent with the expert consensus CBC reference intervals (*p* < 0.05). Compared with the other reference intervals, the diagnostic value of the reference screening interval established was significantly higher in this study (*p* < 0.05). The reference range established is verified by 20 independent samples from 2022, and the results are acceptable.

**Conclusion:**

This study preliminarily established reference intervals for complete blood counts of healthy adults at high altitudes in the Ali region of Tibet to provide a reference point for understanding routine blood markers in permanent residents of high-altitude environments and illustrate the need for regular establishment of laboratory reference intervals on a regular basis.

## Introduction

With an average altitude of 4,300 m, the Ali region of Tibet is one of the highest-altitude areas inhabited by humans. As we all know, as the altitude increases, the air pressure gradually decreases, leading to a corresponding decrease in the oxygen content. When a person goes from low to high altitude, the human body produces a series of acute and chronic compensatory responses to adapt to survive, with various effects on human physiology, which may be the cause of many diseases in the human body (Miehe et al., 2019). Usually, acute acclimatization is characterized by symptoms such as increased respiratory rate, increased heart rate, changes in blood composition (decrease in carbon dioxide concentration), acute mountain sickness (headache, insomnia, fatigue, etc.), and increased metabolic rate. Chronic acclimatization is characterized by increased red blood cell counts, spleen enlargement, thickening of the left ventricular wall, increased metabolic efficiency, and adaptive behavioral changes (e.g., reduced high-intensity activity) (Hui et al., 2016).

In 2021, Tremblay et al. used a geographic information system (GIS)-based approach to quantify the human population at 500-m elevation intervals for each country, and the result found that there are 500.3 million humans living at ≥1,500 m and 14.4 million at ≥3,500 m, while China has the largest absolute population at ≥3,500 m, based on georeferenced data for population (Miehe et al., 2019) and elevation (Global Multiresolution Terrain Elevation Data) (Tremblay and Ainslie, 2021).

Under hypoxic conditions, the most significant manifestations of the hematological system are erythrocytosis due to overcompensation of red blood cells, greater width of leukocyte distribution due to stressful changes in the body’s immune system, and a decrease in the reactive number of platelets (Erelew et al., 1994). Acute high-altitude exposure may have a more pronounced effect on erythrocyte and hemoglobin indices (McDonald et al., 1991). Prolonged exposure to high altitude is associated with a range of alterations in hematological indicators, including erythrocytosis, elevated or lowered platelets, hypercholesterolemia, hyperlipidemia, and the development of hyperuricemia (Beguin, 1999). Ortiz-Prado et al. studied the hematological and lipid parameters and how humans chronically exposed to high altitudes become more fit to function under hypoxic conditions, and the higher the number of RBCs, the easier the oxygen transport, translating into a reduced cardiac output among adapted populations (Ortiz-Prado et al., 2021). These changes in the peripheral blood of the human body have had varying degrees of impact on changes in blood viscosity, human immune function, endothelial cell damage, and venous thrombosis (Ranucci et al., 2015; Boos et al., 2012).

Blood routine analysis is the most commonly used detection index in clinical experiments, and the reference intervals of complete blood count (CBC) reported are essential for the diagnosis and treatment of patients in clinical laboratories. The CBC results obtained at normal sea level under standard atmospheric pressure are not suitable for residents who have lived a long time in high-altitude areas, and the references should change with altitude (Wu et al., 2005; Zhang et al., 2020; Tansey, 2008). The Ministry of Health of the People’s Republic of China published the health industry standard of the People’s Republic of China for reference intervals for blood cell analysis (WS/T 405-2012) according to the guidelines of the Clinical and Laboratory Standards Institute (CLSI) and the International Federation of Clinical Chemistry and Laboratory Medicine (IFCC) (Lahti et al., 2004; CLSI, 2010; Ichihara and Boyd, 2010). According to the WS/T 405-2012 guidelines, this study aims to explore the normal reference intervals for routine hematological indices in healthy populations living in the Tibet–Ali region.

## Methodology

### Study design, setting, and participants

In this study, we selected the population who underwent medical examinations between January 2019 and December 2021 in the clinical laboratory of Ali Regional People’s Hospital. Through the screening process conducted by the health check-up department, we finally obtained specific information on the blood routine indicators of the health check-up population from seven counties in the Ali region, most of which originated from the Tibetan and Han civil servants permanently residing in the cities of these seven counties. Due to the large differences in population size and gender ratio of civil servants in each region, the ratio of females to males is not exactly 1:1. The health screening section omits the names, districts, and units of the personnel as all populations are drawn exclusively from the civil service.

### Inclusion criteria

The study was conducted among a healthy population of both sexes without any type of comorbidity or chronic disease, between the ages of 18 and 79, who had been residing in high-altitude areas for a minimum of 5 years.

## Exclusion criteria

According to the WS/T 405-2012, the following are excluded: 1) blood system diseases, allergic diseases, respiratory system diseases, urinary system diseases, digestive system diseases, rheumatic diseases, thyroid diseases, parasitic infections, malignant tumors, and hereditary diseases and hypertension; 2) recent surgery or medication; 3) recent blood donation, blood transfusion, or massive blood loss; 4) emaciation and malnutrition; 5) alcoholism and smoking; 6) recent strenuous exercise or heavy physical labor; and 7) sexual physical and chemical damage or long-term exposure to chemical substances.

### Variables and outcomes

Uniform blood collection equipment and hematological testing methods were used for all subjects. Two mL of blood was collected from the veins into an anticoagulated vacuum tube (EDTA-K_2_) and analyzed using Seisen Micron XT-4000i (Seisen Micron Co., LTD., Japan). The quality control was carried out using the instrument’s supporting quality control materials. The reference interval standards of various indicators are in accordance with the Health Industry Standard WS/T 405-2012 of the People’s Republic of China.

There were 19 routine blood indicators, namely, white blood cell (WBC), neutrophil (NEUT), lymphocyte (LYMPH), monocyte (MONO), eosinophil (EO), basophil (BASO), red blood cell (RBC), hemoglobin (HGB), hematocrit (HCT), mean corpuscular volume (MCV), mean hemoglobin content (MCH), mean erythrocyte hemoglobin concentration (MCHC), coefficient of variation of red blood cell distribution width (RDW-CV), standard deviation of red blood cell distribution width (RDW-SD), platelet (PLT), platelet distribution width (PDW), mean platelet volume (MPV), platelet-large cell rate (P-LCR), and thrombocytocrit (PCT).

### Data analysis

The data were tested using the Kolmogorov–Smirnov test for normal distribution, and the data are in non-normal distribution. Descriptive statistics were used to analyze and visualize differences between the two reference ranges. The Mann–Whitney U test was used to compare independent samples, and the percentile method was used for the reference range, with 2.5% and 97.5% considered the lower and upper limits of the reference range. A chi-square test was performed to check the association of categorical variables. The Kappa test was used to assess the consistency of the results for the two reference intervals. The measurement data were described as the mean ± standard deviation (X ± SD), and all statistical analyses accepted significance when *p*-value <0.05. Calculations were completed using the IBM SPSS Statistics for Windows, Version 23.0, released by IBM Corp. in 2014. All the references were managed using EndNote X9.

### Ethical consideration

This study was approved by the Ethics Committee of Ali District People’s Hospital (approval number: 2022004), and the Ethics Committee approved the exemption of informed consent.

## Results

### Establishment of the CBC reference interval

The reference ranges of blood routine indexes of healthy adults at the plateau were compared with the national clinical reference ranges. The clinical reference range comes from the Ali District People’s Hospital laboratory in Ali District, and the national clinical reference range comes from the Chinese adult blood cell analysis reference interval (WS/T 405-2012). According to the laboratory examination, the exclusion criteria are based on the industry standard, and one of the following conditions was met: 1) Glu ≥7.0 mmol/L; 2) white blood cells <3.0 × 10^9^/L or >15.0 × 10^9^/L; 3) hemoglobin <90 g/L; 4) albumin <90 g/L; 5) alanine aminotransferase (ALT) > 80 U/L and aspartate aminotransferase (AST) > 80 U/L; 6) urinary protein or urinary sugar is positive. A total of 4,863 cases, including 3,598 males and 1,265 females, were obtained according to the above criteria.

The results showed that the reference ranges of CBC were significantly different than the current national standard clinical reference ranges in the plateau healthy adults, except PLT (*p* < 0.01). The RBC, HGB, and HCT levels were also significantly higher in men than in women (t = 42.31, 56.48, and 49.20; *p* < 0.001). This result indicates that the CBC references need to be established, and the RBC, HGB, and HCT counts should also be established separately by sex in extremely high-altitude environments, as shown in Table 1.

**TABLE 1:** Reference range of CBC.

Variable	N	X ± sd	2.5%–97.5%	Ngari clinical reference range	National clinical reference range
WBC (10 ^ 9/L)	4,863	6.27 ± 1.53	3.78–9.80^b^	4.00–10.00	3.50–9.50
NEUT (10 ^ 9/L)	4,863	3.43 ± 1.17	1.70–6.25^b^	1.80–6.98	1.80–6.30
LYMPH (10 ^ 9/L)	4,863	2.24 ± 0.62	1.23–3.67^b^	1.26–3.35	1.10–3.20
MONO (10 ^ 9/L)	4,863	0.47 ± 0.15	0.24–0.83^b^	0.29–0.95	0.10–0.60
EO (10 ^ 9/L)	4,863	0.15 ± 0.76	0.02–0.37^b^	0.03–0.59	0.02–0.52
BASO (10 ^ 9/L)	4,863	0.02 ± 0.01	0–0.05^b^	0.01–0.07	0–0.06
RBC (10 ^ 12/L)					
Male	3,598	6.07 ± 0.71^a^	4.95–7.82^b^	3.99–5.70	4.30–5.80
Female	1,265	5.16 ± 0.48	4.39–6.23^b^	3.99–5.70	3.80–5.10
HGB (g/L)					
Male	3,598	194 ± 20^a^	161–243^b^	125–182	130–175
Female	1,265	158 ± 18	114–190^b^	125–182	115–150
HCT (%)					
Male	3,598	53.61 ± 5.72^a^	44.80–67.63^b^	38.19–57.91	40.00–50.00
Female	1,265	44.86 ± 4.41	34.97–54.11^b^	38.19–57.91	35.00–45.00
MCV (fL)	4,863	88.13 ± 5.69	75.38–97.70^b^	81.8–95.5	82.0–100.0
MCH (pg)	4,863	31.70 ± 2.41	25.02–35.14^b^	27.0–32.3	27.0–34.0
MCHC (g/L)	4,863	360 ± 16	328–385^b^	324–350	316–354
RDW-CV (%)	4,863	13.88 ± 1.68	12.10–18.81	12.0–13.6	
RDW-SD (fL)	4,863	43.73 ± 4.05	37.90–53.38	37.1–45.7	
PLT (10^9/L)	4,863	233 ± 62	126–371	100–330	125–350
PDW (fL)	4,863	12.52 ± 2.70	9.00–19.04	10.1–16.1	
MPV (fL)	4,863	10.43 ± 1.37	8.62–12.90	9.3–12.1	
P-LCR (%)	4,863	28.47 ± 8.22	15.00–46.22	18.5–42.3	
PCT (%)	4,863	0.24 ± 0.06	0.14–0.36	0.17–0.32	

Note: data are presented as the means ± standard deviation, and two percentiles of 2.5% and 97.5% are used as the lower and upper limits of the reference range.

^a^ indicates that there were statistical differences in the male and female groups.

^b^ indicates that there were statistical differences in the established reference range and the national clinical reference range.

### Reference interval consistency test

We used the Kappa coefficient test to compare the consistency of the established reference intervals with the current national reference intervals to determine consistency for clinical diagnosis. The results showed that the PLT reference interval was consistent with the national clinical reference range and Ali clinical reference range (*p* > 0.05). The NEUT reference interval was consistent with the Ali clinical reference range (*p* > 0.05). The results for other indicators were less consistent (*p* < 0.05). All kappa results and *p*-values are shown in Table 2. It shows that it is essential for laboratories to establish reference intervals to meet clinical needs on a regular basis.

**TABLE 2:** Comparison of three reference intervals in CBC.

Variable	Kappa _1_	*p-value*	Kappa _2_	*p-value*	Kappa _3_	*p-value*	Kappa	*p-value*
WBC	−0.015	<0.001	0.009	0.002	0.024	<0.001	−0.002	0.273
NEUT	−0.005	0.189	−0.010	0.007	−0.005	0.184	0.000	0.891
LYMPH	−0.015	<0.001	−0.009	0.007	0.006	0.070	0.008	0.002
MONO	−0.040	<0.001	−0.051	<0.001	−0.013	0.001	0.056	<0.001
EO	−0.032	<0.001	0.007	0.011	0.038	<0.001	−0.019	<0.001
BASO	−0.057	<0.001	0.005	<0.001	0.062	<0.001	−0.031	<0.001
RBC								
Male	−0.228	<0.001	−0.208	<0.001	0.021	<0.001	−0.024	<0.001
Female	−0.016	0.007	−0.176	<0.001	−0.158	<0.001	0.217	<0.001
HGB								
Male	−0.240	<0.001	−0.275	<0.001	−0.037	<0.001	0.076	<0.001
Female	−0.038	<0.001	−0.260	<0.001	−0.229	<0.001	0.329	<0.001
HCT								
Male	−0.025	<0.001	−0.243	<0.001	−0.216	<0.001	0.319	<0.001
Female	−0.024	0.003	−0.183	<0.001	−0.165	<0.001	0.222	<0.001
MCV	−0.083	<0.001	−0.062	<0.001	0.023	<0.001	−0.062	<0.001
MCH	−0.172	<0.001	−0.051	<0.001	0.123	<0.001	−0.162	<0.001
MCHC	0.249	<0.001	−0.224	<0.001	0.025	<0.001	−0.031	<0.001
RDW-CV	−0.161	<0.001						
RDW-SD	−0.080	<0.001						
PLT	−0.004	0.216	−0.006	0.055	−0.003	0.407	−0.004	0.090
PDW	−0.132	<0.001						
MPV	−0.115	<0.001						
P-LCR	−0.074	<0.001						
PCT	−0.057	<0.001						

Note: kappa _1_ means chi-squared test in the 2.5%–97.5% reference range and Ngari clinical reference range. Kappa _2_ means chi-squared test in the 2.5%–97.5% reference range and national clinical reference range. Kappa _3_ means chi-squared test in the Ngari clinical reference range and national clinical reference range. Kappa means chi-squared test in the 2.5%–97.5% reference range, Ngari clinical reference range, and national clinical reference range. Cohen’s kappa value <0.20 means a poor agreement, 0.21–0.40 means general consistency, 0.41–0.60 means medium consistency, 0.61–0.80 means stronger consistency, and 0.81–1.00 means strong consistency.

### Reference interval results validation

According to the relevant regulations of WS/T 402 for the evaluation and validation requirements of reference intervals, we randomly collected 20 samples from the samples of medical checkups in hospitals in 2022 and used a small sample size to evaluate the established reference intervals. According to the requirements, no more than two of the 20 samples (or 10% of the test results) exceeded the initially reported 95% reference limit, which was considered validated. All the test results passed the established reference interval, and it indicated that the reference interval of the blood cell count in this study was applicable to healthy adults in the Ali region, as shown in Table 3.

**TABLE 3:** CBC reference interval of validation results.

Variable	Established reference range	Validation population reference range	Verification result
WBC	3.78–9.80	3.84–9.21	Pass
NEUT	1.70–6.25	1.86–5.76	Pass
LYMPH	1.23–3.67	1.24–2.57	Pass
MONO	0.24–0.83	0.24–0.81	Pass
EO	0.02–0.37	0.02–0.28	Pass
BASO	0–0.05	0–0.05	Pass
RBC			Pass
Male	4.95–7.82	5.32–7.35	Pass
Female	4.39–6.23	4.75–5.44	Pass
HGB			Pass
Male	161–243	162–229	Pass
Female	114–190	128–187	Pass
HCT			Pass
Male	44.80–67.63	46.50–62.78	Pass
Female	34.97–54.11	38.14–48.50	Pass
MCV	75.38–97.70	75.52–95.11	Pass
MCH	25.02–35.14	25.34–35.40	Pass
MCHC	328–385	328–384	Pass
RDW-CV	12.10–18.81	11.90–22.81	Pass
RDW-SD	37.90–53.38	35.52–67.21	Pass
PLT	126–371	126–322	Pass
PDW	9.00–19.04	9.10–15.70	Pass
MPV	8.62–12.90	8.62–11.90	Pass
P-LCR	15.00–46.22	15.00–39.80	Pass
PCT	0.14–0.36	0.16–0.34	Pass

Note: verify the established reference range according to the health industry standard of the People’s Republic of China WS/T 402.

## Discussion

The Ngari region of Tibet is currently one of highest human settlements (Miehe et al., 2019). Long-term living in a hypoxic environment will produce adaptive changes in blood-related physiological indicators. Changes in CBC have been associated with various physiological and pathological processes in the human body, such as inflammation, tumors, and heart disease (Stellingwerff, et al., 2019; Doutreleau, 2021; Schmidt, 2002; Lafuente et al., 2016). Our study is the latest to discuss changes in reference ranges for hematological parameters in high-altitude populations.

The results show that the RBC, HGB, and HCT of healthy adults were significantly higher in high-altitude areas than in low-altitude areas, and these parameters were higher in males than in females. We found that RBC, HGB, and HCT were elevated in residents at very high altitudes, meaning that the hematopoietic system has been in a state of metabolic stress and has not been alleviated over time in order to adapt to the environment and maintain health.

In the study by Ortiz-Prado et al., oxygen partial pressure is an important indicator in the human body, where the partial pressure of oxygen in the alveoli, arterioles, capillaries, and tissues of the human body plays an important role in oxygen supply and metabolism (Tremblay and Ainslie, 2021). Hemoglobin not only acts as a buffer in the blood but also transports oxygen and carbon dioxide in the red blood cells and is an important criterion for the clinical assessment of anemia (Liu et al., 2023; Storz and Bautista, 2022; Yoshida et al., 2019). The RBC, HGB, HCT, and MCHC levels of the residents were significantly higher than the normal standards at extremely high-altitude areas in this study. The average plateau RBC level was higher than the reference range and close to the critical level of plateau polycythemia. The HGB, HCT, and MCHC levels were also higher than the upper limit of the reference range, suggesting that the residents living in high altitudes still have compensatory high HGB and red blood cell counts to maintain oxygenation of body tissues at very high altitudes. The average blood oxygen saturation was between 87% and 93%, and more RBCs were needed to transport oxygen in people at high altitudes (Kang et al., 2016; Kiers et al., 2019; Konya et al., 2018; Wang et al., 2022). The results suggest that humans have changed the compensation mechanism to better adapt to life at extremely high altitudes.

We can observe that the reference range of PLT was wider than the national clinical reference range. There are some opposite studies, and they concluded that chronic hypoxia leads to decreased PLT and increased MPV, and they suggested that the excessive compensatory proliferation of RBC leads to decreased PLT under hypoxic conditions in their previous study (Erslev, 1992; McDonald, et al., 1991). The proliferation and differentiation of the megakaryocytes increased the number of PLT in the bone marrow under stress, and several studies have suggested that thrombopoietin may play a complex role in regulating hypoxia and hypobaric exposure (Kaupke et al., 1993; Qi et al., 2017). In summary, numerous current studies support the conclusion that PLT reactivity is enhanced under the high-altitude hypoxic environment, and its mechanism may be related to the enhancement of Ca^2+^ levels and calpain activity, the change of blood viscosity, and the damage of endothelial cells under a hypoxic environment (Kheifetz and Scholz, 2019). Epidemiological data suggested that there is an increased risk of thrombotic events and various thrombotic disorders associated with exposure to high altitudes (Mantysaari et al., 2011; Ranucci et al., 2015; Tyagi et al., 2014). We support that the level of high PLT may increase the risk of thrombosis in high-altitude populations.

In terms of WBC lines, the results showed that the reference ranges for lymphocytes and monocytes were higher than the national clinical reference ranges. This suggests that there is more immune stress at high altitudes. We found that the increase in lymphocytes and monocytes may be caused by immune adaptation to hypoxia, and hypoxia affects the proliferation, differentiation, and apoptosis of immune cells, such as the hypoxia sensitivity of lymphocyte membrane K channel genes (Boos, et al., 2012; Smallman, et al., 2011). In addition, it can also regulate the expression of related genes, enhance intracellular glycolysis, affect the energy supply of cells, and ultimately lead to decreased immunity (Conforti et al., 2003; Sitkovsky and Lukashev, 2005).

In summary, this study established a reference interval for a complete blood count of healthy adults in Ngari, Tibet, which was in line with the existing health status of the local population, and passed the verification of the reference interval based on this result. Our study provided a reference standard for diagnosing diseases and effectively reduced the rate of missed diagnoses in plateau residents. Clinical laboratories ought to establish their bespoke laboratory reference ranges, thereby facilitating clinicians in promptly and precisely assessing the health status of their patients.

## Limitations

The current study has some major limitations. First, our study was retrospective, and the ratio of men to women in this study did not match. Second, the physical examination population in this study came from only one hospital, so there may be selection bias. Therefore, multi-center data studies are needed to obtain stable and consistent reference intervals, and further work is needed to verify the applicability to other high-altitude populations around the world. Third, the reference interval for CBC in healthy adults at very high altitudes should be continuously optimized, necessitating more evidence-based clinical interpretations at other extremely high altitudes.

## Conclusions

This study established the reference standards for 13 parameters of the XT-1800i system for a cohort of healthy adults living in the Ngari region of Tibet for a long time. This is the first study to report the concentration profiles of routine blood parameters on the XT-1800i system in relation to health in high-altitude healthy adults. These data have initially established the reference ranges of blood parameters for healthy adults at very high altitudes for the first time, providing clinicians in the Tibet Ali region with reliable reference intervals for blood cell analysis and improving the accuracy of clinicians’ diagnosis of diseases.

## Data Availability

The raw data supporting the conclusions of this article will be made available by the authors, without undue reservation.
